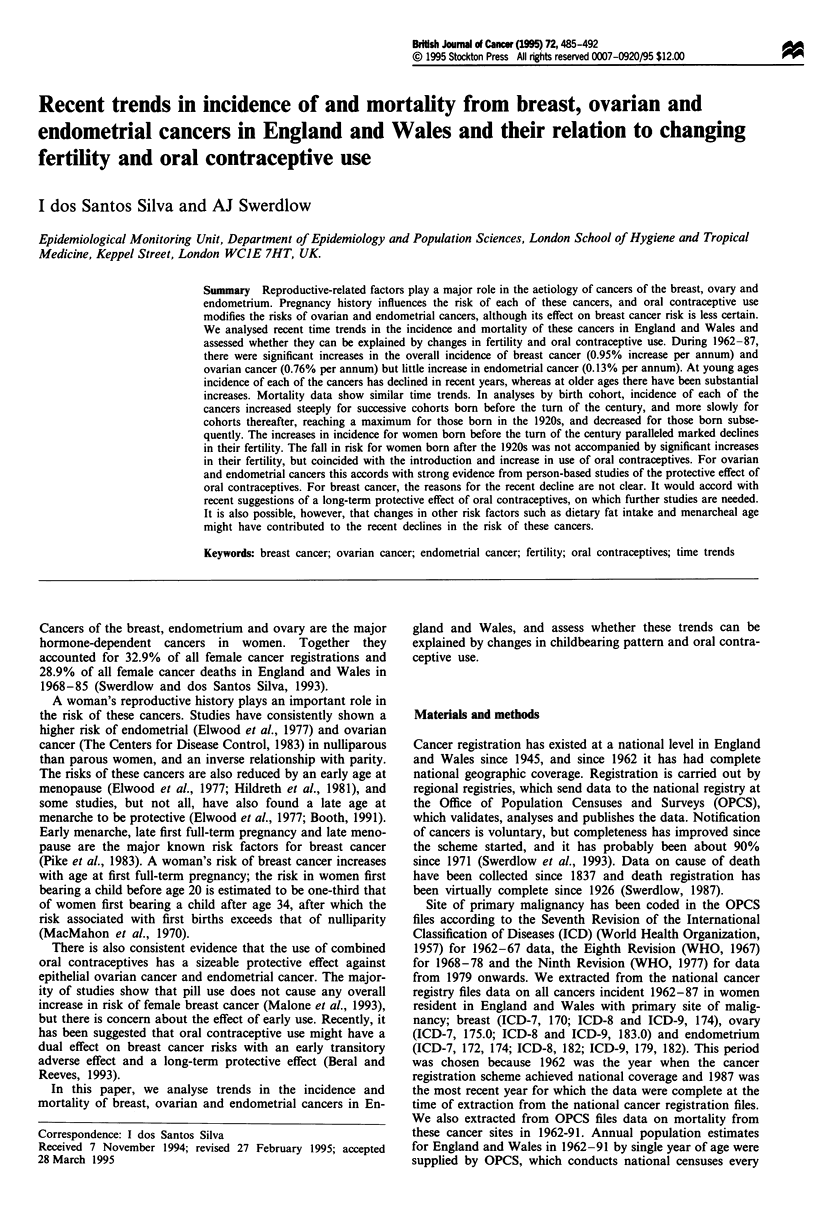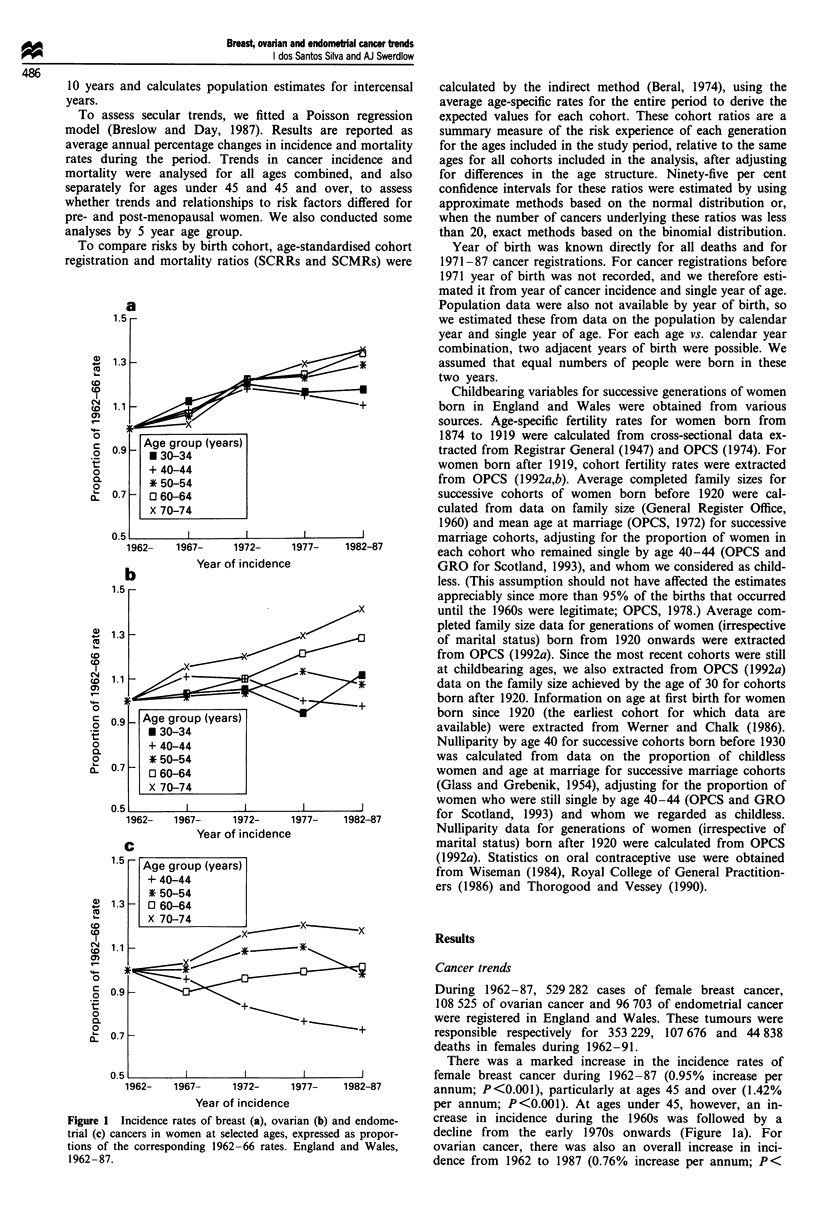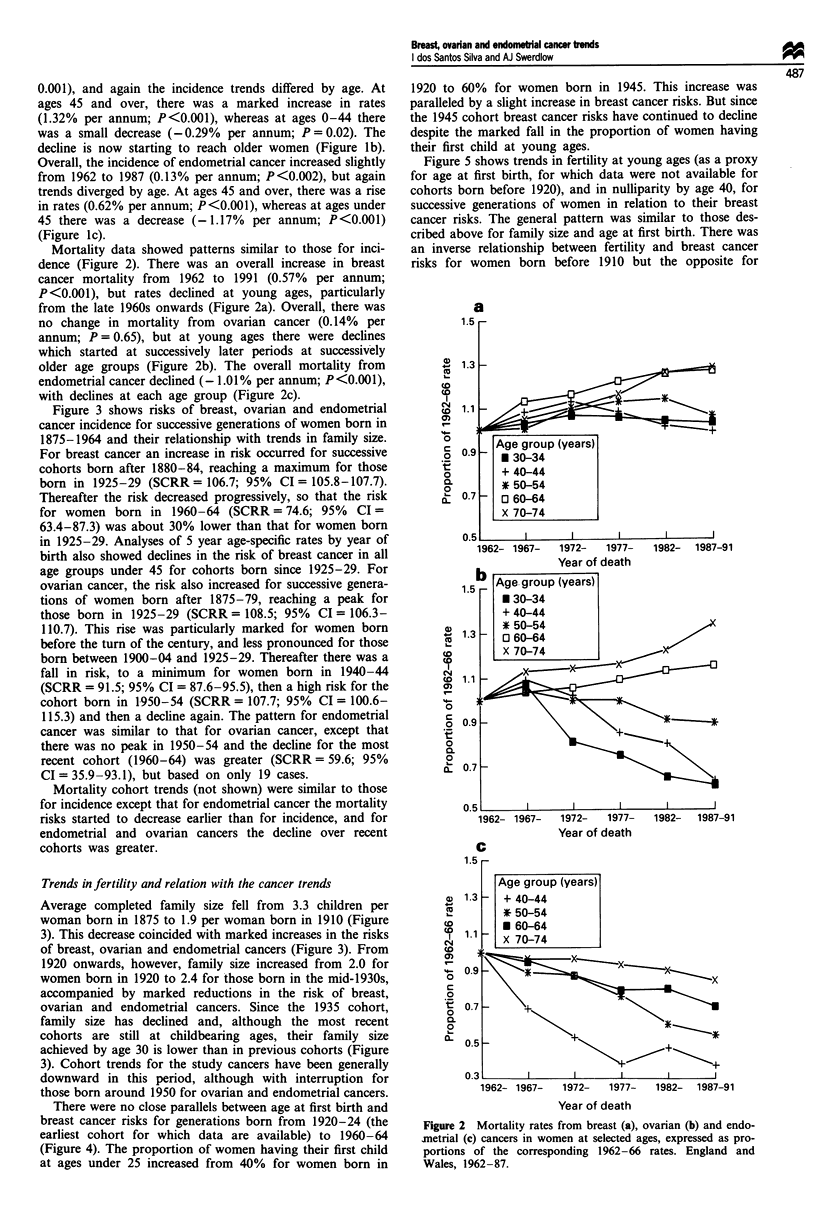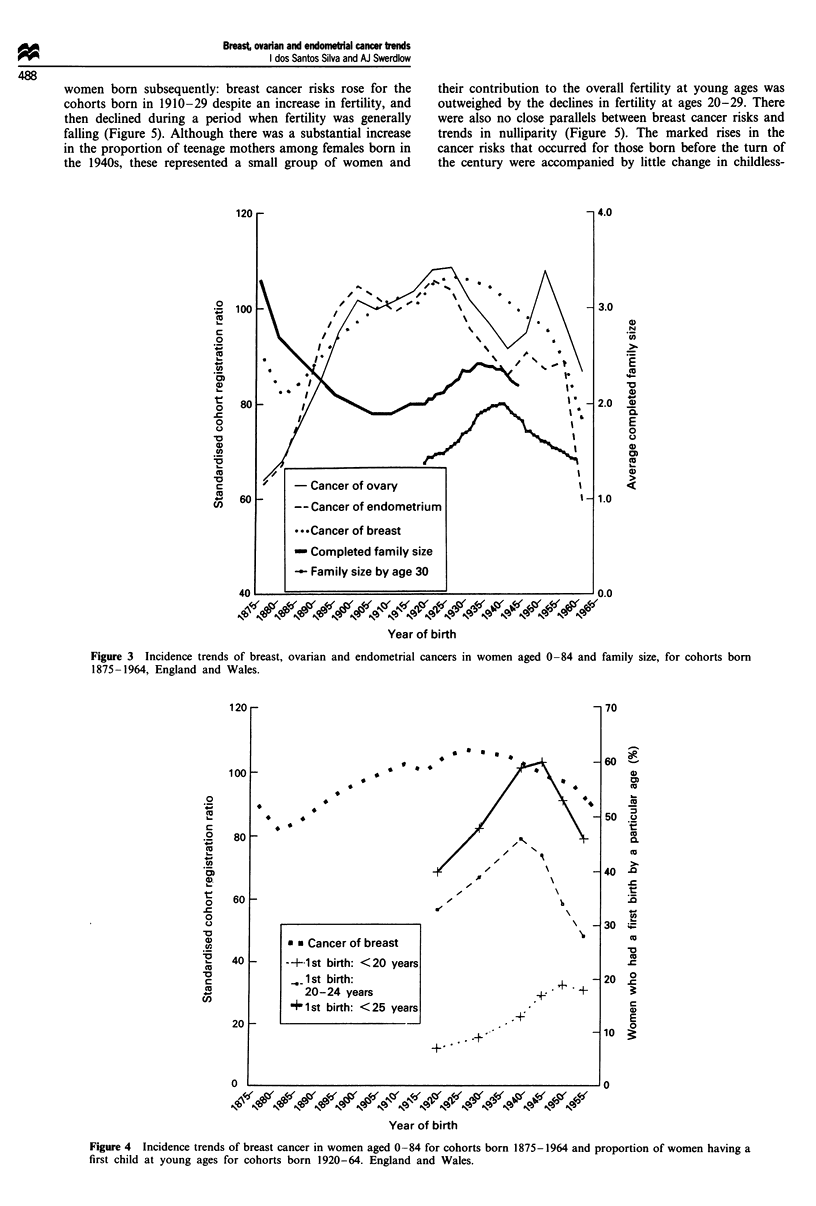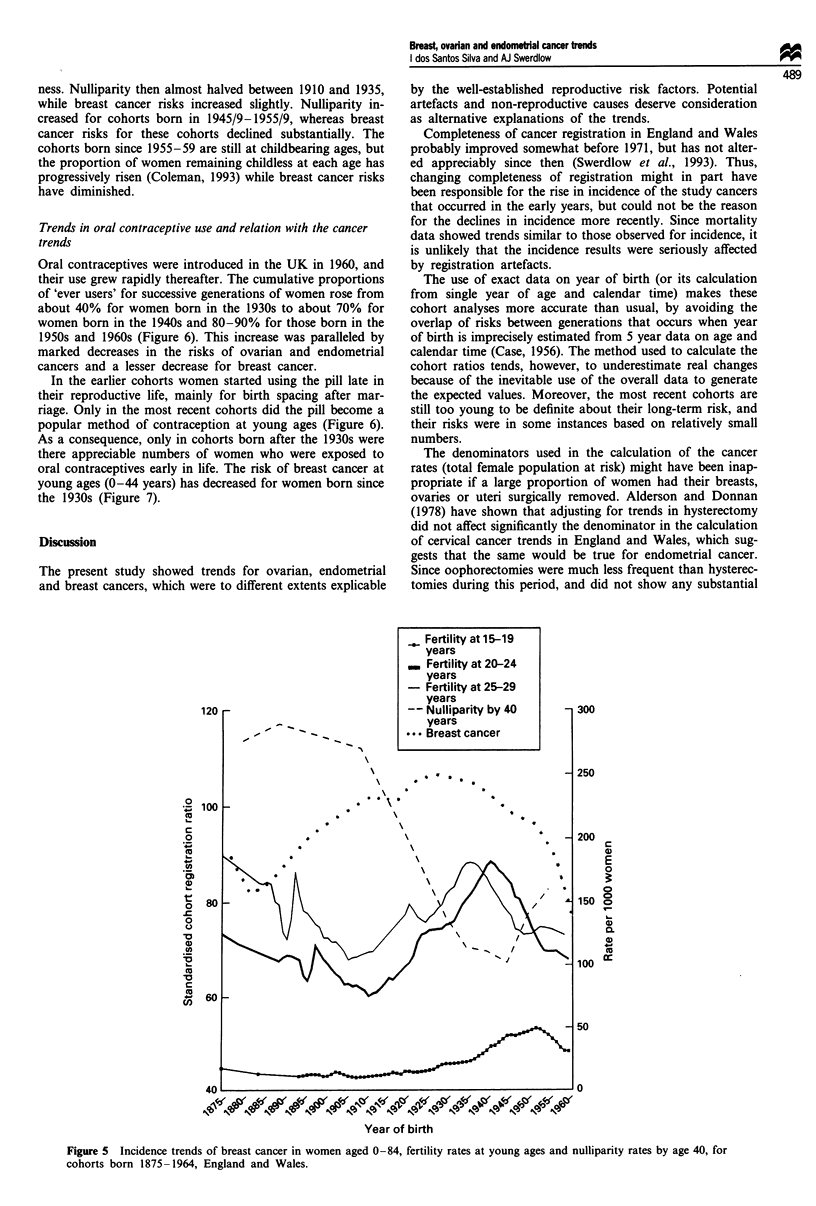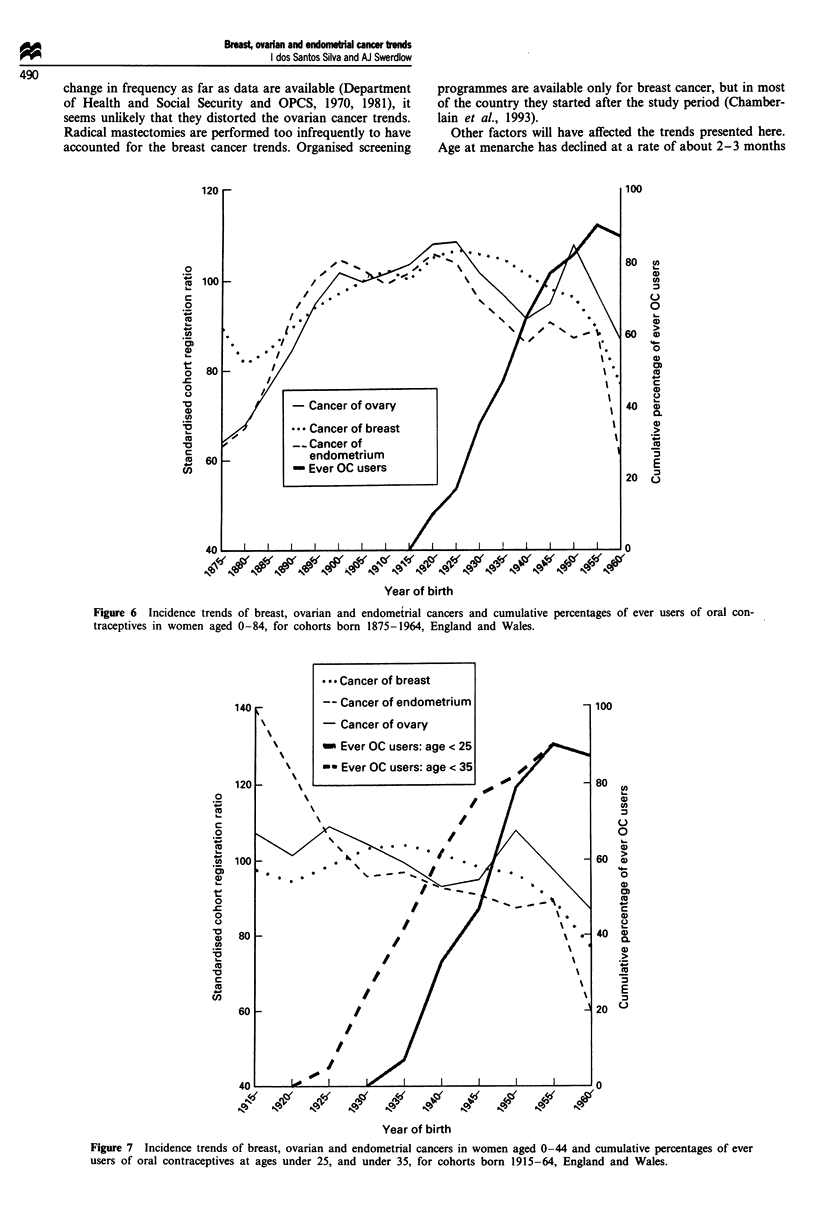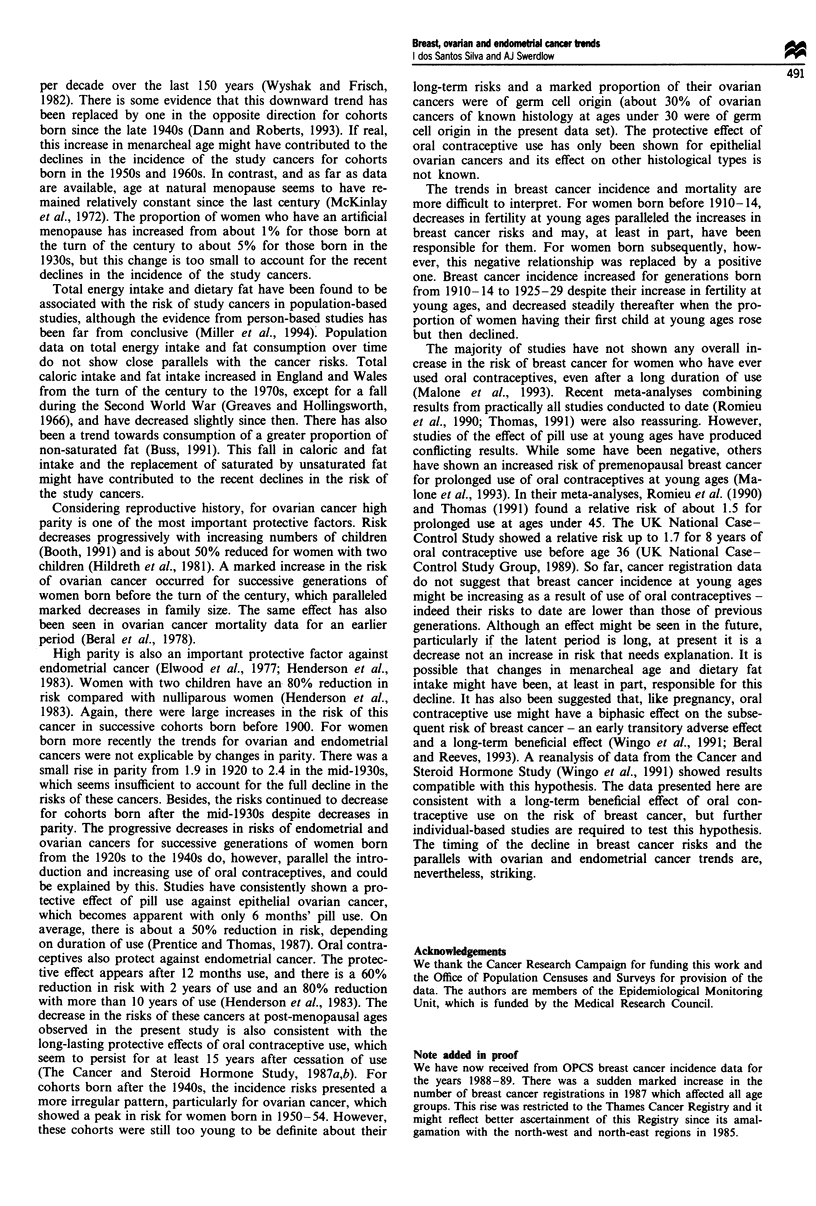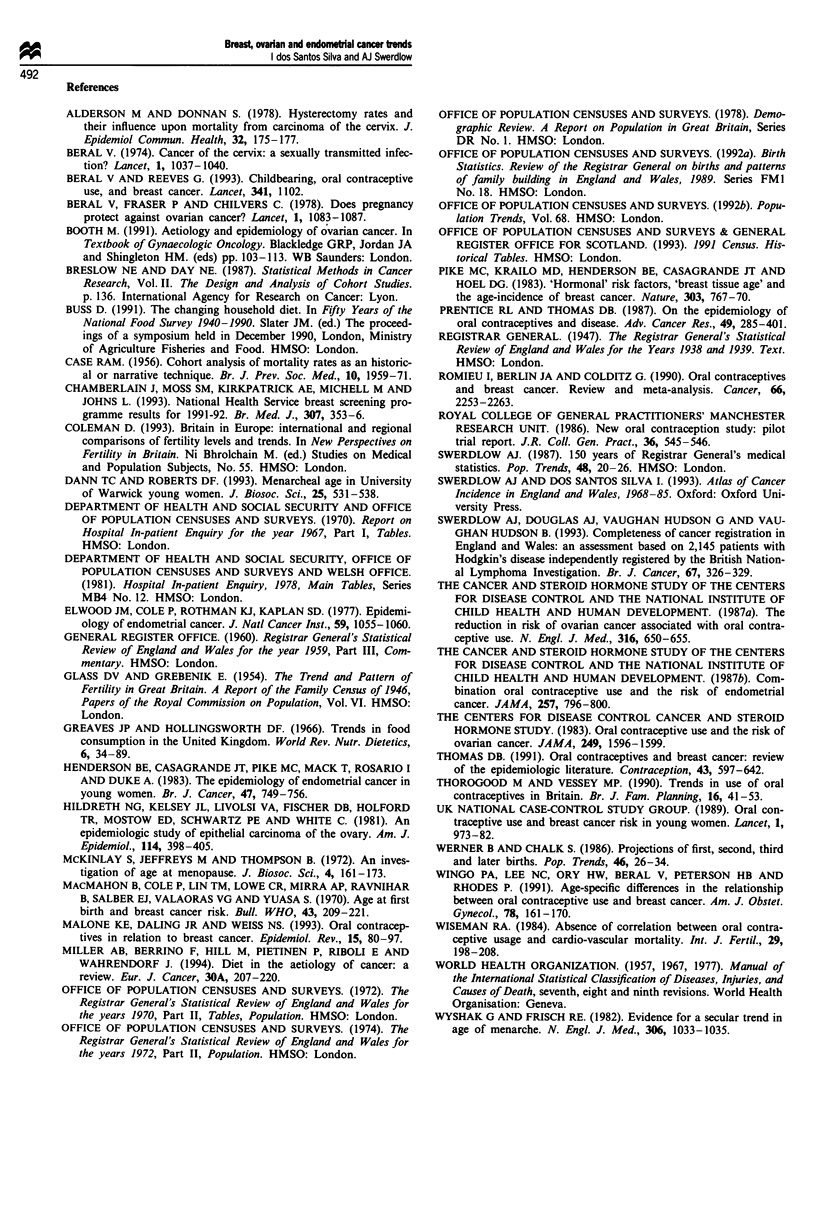# Recent trends in incidence of and mortality from breast, ovarian and endometrial cancers in England and Wales and their relation to changing fertility and oral contraceptive use.

**DOI:** 10.1038/bjc.1995.360

**Published:** 1995-08

**Authors:** I. dos Santos Silva, A. J. Swerdlow

**Affiliations:** Department of Epidemiology and Population Sciences, London School of Hygiene and Tropical Medicine, UK.

## Abstract

Reproductive-related factors play a major role in the aetiology of cancers of the breast, ovary and endometrium. Pregnancy history influences the risk of each of these cancers, and oral contraceptive use modifies the risks of ovarian and endometrial cancers, although its effect on breast cancer risk is less certain. We analysed recent time trends in the incidence and mortality of these cancers in England and Wales and assessed whether they can be explained by changes in fertility and oral contraceptive use. During 1962-87, there were significant increases in the overall incidence of breast cancer (0.95% increase per annum) and ovarian cancer (0.76% per annum) but little increase in endometrial cancer (0.13% per annum). At young ages incidence of each of the cancers has declined in recent years, whereas at older ages there have been substantial increases. Mortality data show similar time trends. In analyses by birth cohort, incidence of each of the cancers increased steeply for successive cohorts born before the turn of the century, and more slowly for cohorts thereafter, reaching a maximum for those born in the 1920s, and decreased for those born subsequently. The increases in incidence for women born before the turn of the century paralleled marked declines in their fertility. The fall in risk for women born after the 1920s was not accompanied by significant increases in their fertility, but coincided with the introduction and increase in use of oral contraceptives. For ovarian and endometrial cancers this accords with strong evidence from person-based studies of the protective effect of oral contraceptives. For breast cancer, the reasons for the recent decline are not clear. It would accord with recent suggestions of a long-term protective effect of oral contraceptives, on which further studies are needed. It is also possible, however, that changes in other risk factors such as dietary fat intake and menarcheal age might have contributed to the recent declines in the risk of these cancers.


British Journal of Cancer (1995) 72, 485-492
© 1995 Stockton Press All rights reserved 0007-0920/95 $12.00

### Recent trends in incidence of and mortality from breast, ovarian and endometrial cancers in England and Wales and their relation to changing fertility and oral contraceptive use

I dos Santos Silva and AJ Swerdlow

*Epidemiological Monitoring Unit, Department of Epidemiology and Population Sciences, London School of Hygiene and Tropical Medicine, Keppel Street, London WC1E 7HT, UK.*

**Summary** Reproductive-related factors play a major role in the aetiology of cancers of the breast, ovary and endometrium. Pregnancy history influences the risk of each of these cancers, and oral contraceptive use modifies the risks of ovarian and endometrial cancers, although its effect on breast cancer risk is less certain. We analysed recent time trends in the incidence and mortality of these cancers in England and Wales and assessed whether they can be explained by changes in fertility and oral contraceptive use. During 1962-87, there were significant increases in the overall incidence of breast cancer (0.95% increase per annum) and ovarian cancer (0.76% per annum) but little increase in endometrial cancer (0.13% per annum). At young ages incidence of each of the cancers has declined in recent years, whereas at older ages there have been substantial increases. Mortality data show similar time trends. In analyses by birth cohort, incidence of each of the cancers increased steeply for successive cohorts born before the turn of the century, and more slowly for cohorts thereafter, reaching a maximum for those born in the 1920s, and decreased for those born subsequently. The increases in incidence for women born before the turn of the century paralleled marked declines in their fertility. The fall in risk for women born after the 1920s was not accompanied by significant increases in their fertility, but coincided with the introduction and increase in use of oral contraceptives. For ovarian and endometrial cancers this accords with strong evidence from person-based studies of the protective effect of oral contraceptives. For breast cancer, the reasons for the recent decline are not clear. It would accord with recent suggestions of a long-term protective effect of oral contraceptives, on which further studies are needed. It is also possible, however, that changes in other risk factors such as dietary fat intake and menarcheal age might have contributed to the recent declines in the risk of these cancers.

**Keywords:** breast cancer; ovarian cancer; endometrial cancer; fertility; oral contraceptives; time trends

Cancers of the breast, endometrium and ovary are the major hormone-dependent cancers in women. Together they accounted for 32.9% of all female cancer registrations and 28.9% of all female cancer deaths in England and Wales in 1968-85 (Swerdlow and dos Santos Silva, 1993).

A woman's reproductive history plays an important role in the risk of these cancers. Studies have consistently shown a higher risk of endometrial (Elwood *et al.*, 1977) and ovarian cancer (The Centers for Disease Control, 1983) in nulliparous than parous women, and an inverse relationship with parity. The risks of these cancers are also reduced by an early age at menopause (Elwood *et al.*, 1977; Hildreth *et al.*, 1981), and some studies, but not all, have also found a late age at menarche to be protective (Elwood *et al.*, 1977; Booth, 1991). Early menarche, late first full-term pregnancy and late menopause are the major known risk factors for breast cancer (Pike *et al.*, 1983). A woman's risk of breast cancer increases with age at first full-term pregnancy; the risk in women first bearing a child before age 20 is estimated to be one-third that of women first bearing a child after age 34, after which the risk associated with first births exceeds that of nulliparity (MacMahon *et al.*, 1970).

There is also consistent evidence that the use of combined oral contraceptives has a sizeable protective effect against epithelial ovarian cancer and endometrial cancer. The majority of studies show that pill use does not cause any overall increase in risk of female breast cancer (Malone *et al.*, 1993), but there is concern about the effect of early use. Recently, it has been suggested that oral contraceptive use might have a dual effect on breast cancer risks with an early transitory adverse effect and a long-term protective effect (Beral and Reeves, 1993).

In this paper, we analyse trends in the incidence and mortality of breast, ovarian and endometrial cancers in England and Wales, and assess whether these trends can be explained by changes in childbearing pattern and oral contraceptive use.

Correspondence: I dos Santos Silva
Received 7 November 1994; revised 27 February 1995; accepted 28 March 1995

### Materials and methods

Cancer registration has existed at a national level in England and Wales since 1945, and since 1962 it has had complete national geographic coverage. Registration is carried out by regional registries, which send data to the national registry at the Office of Population Censuses and Surveys (OPCS), which validates, analyses and publishes the data. Notification of cancers is voluntary, but completeness has improved since the scheme started, and it has probably been about 90% since 1971 (Swerdlow *et al.*, 1993). Data on cause of death have been collected since 1837 and death registration has been virtually complete since 1926 (Swerdlow, 1987).

Site of primary malignancy has been coded in the OPCS files according to the Seventh Revision of the International Classification of Diseases (ICD) (World Health Organization, 1957) for 1962-67 data, the Eighth Revision (WHO, 1967) for 1968-78 and the Ninth Revision (WHO, 1977) for data from 1979 onwards. We extracted from the national cancer registry files data on all cancers incident 1962-87 in women resident in England and Wales with primary site of malignancy; breast (ICD-7, 170; ICD-8 and ICD-9, 174), ovary (ICD-7, 175.0; ICD-8 and ICD-9, 183.0) and endometrium (ICD-7, 172, 174; ICD-8, 182; ICD-9, 179, 182). This period was chosen because 1962 was the year when the cancer registration scheme achieved national coverage and 1987 was the most recent year for which the data were complete at the time of extraction from the national cancer registration files. We also extracted from OPCS files data on mortality from these cancer sites in 1962-91. Annual population estimates for England and Wales in 1962-91 by single year of age were supplied by OPCS, which conducts national censuses every

10 years and calculates population estimates for intercensal years.

To assess secular trends, we fitted a Poisson regression model (Breslow and Day, 1987). Results are reported as average annual percentage changes in incidence and mortality rates during the period. Trends in cancer incidence and mortality were analysed for all ages combined, and also separately for ages under 45 and 45 and over, to assess whether trends and relationships to risk factors differed for pre- and post-menopausal women. We also conducted some analyses by 5 year age group.

To compare risks by birth cohort, age-standardised cohort registration and mortality ratios (SCRRs and SCMRs) were calculated by the indirect method (Beral, 1974), using the average age-specific rates for the entire period to derive the expected values for each cohort. These cohort ratios are a summary measure of the risk experience of each generation for the ages included in the study period, relative to the same ages for all cohorts included in the analysis, after adjusting for differences in the age structure. Ninety-five per cent confidence intervals for these ratios were estimated by using approximate methods based on the normal distribution or, when the number of cancers underlying these ratios was less than 20, exact methods based on the binomial distribution.

Year of birth was known directly for all deaths and for 1971–87 cancer registrations. For cancer registrations before 1971 year of birth was not recorded, and we therefore estimated it from year of cancer incidence and single year of age. Population data were also not available by year of birth, so we estimated these from data on the population by calendar year and single year of age. For each age *vs.* calendar year combination, two adjacent years of birth were possible. We assumed that equal numbers of people were born in these two years.

Childbearing variables for successive generations of women born in England and Wales were obtained from various sources. Age-specific fertility rates for women born from 1874 to 1919 were calculated from cross-sectional data extracted from Registrar General (1947) and OPCS (1974). For women born after 1919, cohort fertility rates were extracted from OPCS (1992*a*,*b*). Average completed family sizes for successive cohorts of women born before 1920 were calculated from data on family size (General Register Office, 1960) and mean age at marriage (OPCS, 1972) for successive marriage cohorts, adjusting for the proportion of women in each cohort who remained single by age 40–44 (OPCS and GRO for Scotland, 1993), and whom we considered as childless. (This assumption should not have affected the estimates appreciably since more than 95% of the births that occurred until the 1960s were legitimate; OPCS, 1978.) Average completed family size data for generations of women (irrespective of marital status) born from 1920 onwards were extracted from OPCS (1992*a*). Since the most recent cohorts were still at childbearing ages, we also extracted from OPCS (1992*a*) data on the family size achieved by the age of 30 for cohorts born after 1920. Information on age at first birth for women born since 1920 (the earliest cohort for which data are available) were extracted from Werner and Chalk (1986). Nulliparity by age 40 for successive cohorts born before 1930 was calculated from data on the proportion of childless women and age at marriage for successive marriage cohorts (Glass and Grebenik, 1954), adjusting for the proportion of women who were still single by age 40–44 (OPCS and GRO for Scotland, 1993) and whom we regarded as childless. Nulliparity data for generations of women (irrespective of marital status) born after 1920 were calculated from OPCS (1992*a*). Statistics on oral contraceptive use were obtained from Wiseman (1984), Royal College of General Practitioners (1986) and Thorogood and Vessey (1990).

**Figure 1** Incidence rates of breast (**a**), ovarian (**b**) and endometrial (**c**) cancers in women at selected ages, expressed as proportions of the corresponding 1962–66 rates. England and Wales, 1962–87.

### Results

### *Cancer trends*

During 1962–87, 529 282 cases of female breast cancer, 108 525 of ovarian cancer and 96 703 of endometrial cancer were registered in England and Wales. These tumours were responsible respectively for 353 229, 107 676 and 44 838 deaths in females during 1962–91.

There was a marked increase in the incidence rates of female breast cancer during 1962–87 (0.95% increase per annum; $P<0.001$), particularly at ages 45 and over (1.42% per annum; $P<0.001$). At ages under 45, however, an increase in incidence during the 1960s was followed by a decline from the early 1970s onwards (Figure 1a). For ovarian cancer, there was also an overall increase in incidence from 1962 to 1987 (0.76% increase per annum; $P<$

0.001), and again the incidence trends differed by age. At ages 45 and over, there was a marked increase in rates (1.32% per annum; $P<0.001$), whereas at ages 0–44 there was a small decrease (−0.29% per annum; $P=0.02$). The decline is now starting to reach older women (Figure 1b). Overall, the incidence of endometrial cancer increased slightly from 1962 to 1987 (0.13% per annum; $P<0.002$), but again trends diverged by age. At ages 45 and over, there was a rise in rates (0.62% per annum; $P<0.001$), whereas at ages under 45 there was a decrease (−1.17% per annum; $P<0.001$) (Figure 1c).

Mortality data showed patterns similar to those for incidence (Figure 2). There was an overall increase in breast cancer mortality from 1962 to 1991 (0.57% per annum; $P<0.001$), but rates declined at young ages, particularly from the late 1960s onwards (Figure 2a). Overall, there was no change in mortality from ovarian cancer (0.14% per annum; $P=0.65$), but at young ages there were declines which started at successively later periods at successively older age groups (Figure 2b). The overall mortality from endometrial cancer declined (−1.01% per annum; $P<0.001$), with declines at each age group (Figure 2c).

Figure 3 shows risks of breast, ovarian and endometrial cancer incidence for successive generations of women born in 1875–1964 and their relationship with trends in family size. For breast cancer an increase in risk occurred for successive cohorts born after 1880–84, reaching a maximum for those born in 1925–29 (SCRR = 106.7; 95% CI = 105.8–107.7). Thereafter the risk decreased progressively, so that the risk for women born in 1960–64 (SCRR = 74.6; 95% CI = 63.4–87.3) was about 30% lower than that for women born in 1925–29. Analyses of 5 year age-specific rates by year of birth also showed declines in the risk of breast cancer in all age groups under 45 for cohorts born since 1925–29. For ovarian cancer, the risk also increased for successive generations of women born after 1875–79, reaching a peak for those born in 1925–29 (SCRR = 108.5; 95% CI = 106.3–110.7). This rise was particularly marked for women born before the turn of the century, and less pronounced for those born between 1900–04 and 1925–29. Thereafter there was a fall in risk, to a minimum for women born in 1940–44 (SCRR = 91.5; 95% CI = 87.6–95.5), then a high risk for the cohort born in 1950–54 (SCRR = 107.7; 95% CI = 100.6–115.3) and then a decline again. The pattern for endometrial cancer was similar to that for ovarian cancer, except that there was no peak in 1950–54 and the decline for the most recent cohort (1960–64) was greater (SCRR = 59.6; 95% CI = 35.9–93.1), but based on only 19 cases.

Mortality cohort trends (not shown) were similar to those for incidence except that for endometrial cancer the mortality risks started to decrease earlier than for incidence, and for endometrial and ovarian cancers the decline over recent cohorts was greater.

### *Trends in fertility and relation with the cancer trends*

Average completed family size fell from 3.3 children per woman born in 1875 to 1.9 per woman born in 1910 (Figure 3). This decrease coincided with marked increases in the risks of breast, ovarian and endometrial cancers (Figure 3). From 1920 onwards, however, family size increased from 2.0 for women born in 1920 to 2.4 for those born in the mid-1930s, accompanied by marked reductions in the risk of breast, ovarian and endometrial cancers. Since the 1935 cohort, family size has declined and, although the most recent cohorts are still at childbearing ages, their family size achieved by age 30 is lower than in previous cohorts (Figure 3). Cohort trends for the study cancers have been generally downward in this period, although with interruption for those born around 1950 for ovarian and endometrial cancers.

There were no close parallels between age at first birth and breast cancer risks for generations born from 1920–24 (the earliest cohort for which data are available) to 1960–64 (Figure 4). The proportion of women having their first child at ages under 25 increased from 40% for women born in 1920 to 60% for women born in 1945. This increase was paralleled by a slight increase in breast cancer risks. But since the 1945 cohort breast cancer risks have continued to decline despite the marked fall in the proportion of women having their first child at young ages.

Figure 5 shows trends in fertility at young ages (as a proxy for age at first birth, for which data were not available for cohorts born before 1920), and in nulliparity by age 40, for successive generations of women in relation to their breast cancer risks. The general pattern was similar to those described above for family size and age at first birth. There was an inverse relationship between fertility and breast cancer risks for women born before 1910 but the opposite for

**Figure 2** Mortality rates from breast (**a**), ovarian (**b**) and endometrial (**c**) cancers in women at selected ages, expressed as proportions of the corresponding 1962–66 rates. England and Wales, 1962–87.

women born subsequently: breast cancer risks rose for the cohorts born in 1910–29 despite an increase in fertility, and then declined during a period when fertility was generally falling (Figure 5). Although there was a substantial increase in the proportion of teenage mothers among females born in the 1940s, these represented a small group of women and their contribution to the overall fertility at young ages was outweighed by the declines in fertility at ages 20–29. There were also no close parallels between breast cancer risks and trends in nulliparity (Figure 5). The marked rises in the cancer risks that occurred for those born before the turn of the century were accompanied by little change in childless-

**Figure 3** Incidence trends of breast, ovarian and endometrial cancers in women aged 0–84 and family size, for cohorts born 1875–1964, England and Wales.

**Figure 4** Incidence trends of breast cancer in women aged 0–84 for cohorts born 1875–1964 and proportion of women having a first child at young ages for cohorts born 1920–64. England and Wales.

ness. Nulliparity then almost halved between 1910 and 1935, while breast cancer risks increased slightly. Nulliparity increased for cohorts born in 1945/9–1955/9, whereas breast cancer risks for these cohorts declined substantially. The cohorts born since 1955–59 are still at childbearing ages, but the proportion of women remaining childless at each age has progressively risen (Coleman, 1993) while breast cancer risks have diminished.

### *Trends in oral contraceptive use and relation with the cancer trends*

Oral contraceptives were introduced in the UK in 1960, and their use grew rapidly thereafter. The cumulative proportions of 'ever users' for successive generations of women rose from about 40% for women born in the 1930s to about 70% for women born in the 1940s and 80–90% for those born in the 1950s and 1960s (Figure 6). This increase was paralleled by marked decreases in the risks of ovarian and endometrial cancers and a lesser decrease for breast cancer.

In the earlier cohorts women started using the pill late in their reproductive life, mainly for birth spacing after marriage. Only in the most recent cohorts did the pill become a popular method of contraception at young ages (Figure 6). As a consequence, only in cohorts born after the 1930s were there appreciable numbers of women who were exposed to oral contraceptives early in life. The risk of breast cancer at young ages (0–44 years) has decreased for women born since the 1930s (Figure 7).

### Discussion

The present study showed trends for ovarian, endometrial and breast cancers, which were to different extents explicable by the well-established reproductive risk factors. Potential artefacts and non-reproductive causes deserve consideration as alternative explanations of the trends.

Completeness of cancer registration in England and Wales probably improved somewhat before 1971, but has not altered appreciably since then (Swerdlow *et al.*, 1993). Thus, changing completeness of registration might in part have been responsible for the rise in incidence of the study cancers that occurred in the early years, but could not be the reason for the declines in incidence more recently. Since mortality data showed trends similar to those observed for incidence, it is unlikely that the incidence results were seriously affected by registration artefacts.

The use of exact data on year of birth (or its calculation from single year of age and calendar time) makes these cohort analyses more accurate than usual, by avoiding the overlap of risks between generations that occurs when year of birth is imprecisely estimated from 5 year data on age and calendar time (Case, 1956). The method used to calculate the cohort ratios tends, however, to underestimate real changes because of the inevitable use of the overall data to generate the expected values. Moreover, the most recent cohorts are still too young to be definite about their long-term risk, and their risks were in some instances based on relatively small numbers.

The denominators used in the calculation of the cancer rates (total female population at risk) might have been inappropriate if a large proportion of women had their breasts, ovaries or uteri surgically removed. Alderson and Donnan (1978) have shown that adjusting for trends in hysterectomy did not affect significantly the denominator in the calculation of cervical cancer trends in England and Wales, which suggests that the same would be true for endometrial cancer. Since oophorectomies were much less frequent than hysterectomies during this period, and did not show any substantial

**Figure 5** Incidence trends of breast cancer in women aged 0–84, fertility rates at young ages and nulliparity rates by age 40, for cohorts born 1875–1964, England and Wales.

change in frequency as far as data are available (Department of Health and Social Security and OPCS, 1970, 1981), it seems unlikely that they distorted the ovarian cancer trends. Radical mastectomies are performed too infrequently to have accounted for the breast cancer trends. Organised screening programmes are available only for breast cancer, but in most of the country they started after the study period (Chamberlain *et al.*, 1993).

Other factors will have affected the trends presented here. Age at menarche has declined at a rate of about 2–3 months

**Figure 6** Incidence trends of breast, ovarian and endometrial cancers and cumulative percentages of ever users of oral contraceptives in women aged 0–84, for cohorts born 1875–1964, England and Wales.

**Figure 7** Incidence trends of breast, ovarian and endometrial cancers in women aged 0–44 and cumulative percentages of ever users of oral contraceptives at ages under 25, and under 35, for cohorts born 1915–64, England and Wales.

per decade over the last 150 years (Wyshak and Frisch, 1982). There is some evidence that this downward trend has been replaced by one in the opposite direction for cohorts born since the late 1940s (Dann and Roberts, 1993). If real, this increase in menarcheal age might have contributed to the declines in the incidence of the study cancers for cohorts born in the 1950s and 1960s. In contrast, and as far as data are available, age at natural menopause seems to have remained relatively constant since the last century (McKinlay *et al.*, 1972). The proportion of women who have an artificial menopause has increased from about 1% for those born at the turn of the century to about 5% for those born in the 1930s, but this change is too small to account for the recent declines in the incidence of the study cancers.

Total energy intake and dietary fat have been found to be associated with the risk of study cancers in population-based studies, although the evidence from person-based studies has been far from conclusive (Miller *et al.*, 1994). Population data on total energy intake and fat consumption over time do not show close parallels with the cancer risks. Total caloric intake and fat intake increased in England and Wales from the turn of the century to the 1970s, except for a fall during the Second World War (Greaves and Hollingsworth, 1966), and have decreased slightly since then. There has also been a trend towards consumption of a greater proportion of non-saturated fat (Buss, 1991). This fall in caloric and fat intake and the replacement of saturated by unsaturated fat might have contributed to the recent declines in the risk of the study cancers.

Considering reproductive history, for ovarian cancer high parity is one of the most important protective factors. Risk decreases progressively with increasing numbers of children (Booth, 1991) and is about 50% reduced for women with two children (Hildreth *et al.*, 1981). A marked increase in the risk of ovarian cancer occurred for successive generations of women born before the turn of the century, which paralleled marked decreases in family size. The same effect has also been seen in ovarian cancer mortality data for an earlier period (Beral *et al.*, 1978).

High parity is also an important protective factor against endometrial cancer (Elwood *et al.*, 1977; Henderson *et al.*, 1983). Women with two children have an 80% reduction in risk compared with nulliparous women (Henderson *et al.*, 1983). Again, there were large increases in the risk of this cancer in successive cohorts born before 1900. For women born more recently the trends for ovarian and endometrial cancers were not explicable by changes in parity. There was a small rise in parity from 1.9 in 1920 to 2.4 in the mid-1930s, which seems insufficient to account for the full decline in the risks of these cancers. Besides, the risks continued to decrease for cohorts born after the mid-1930s despite decreases in parity. The progressive decreases in risks of endometrial and ovarian cancers for successive generations of women born from the 1920s to the 1940s do, however, parallel the introduction and increasing use of oral contraceptives, and could be explained by this. Studies have consistently shown a protective effect of pill use against epithelial ovarian cancer, which becomes apparent with only 6 months' pill use. On average, there is about a 50% reduction in risk, depending on duration of use (Prentice and Thomas, 1987). Oral contraceptives also protect against endometrial cancer. The protective effect appears after 12 months use, and there is a 60% reduction in risk with 2 years of use and an 80% reduction with more than 10 years of use (Henderson *et al.*, 1983). The decrease in the risks of these cancers at post-menopausal ages observed in the present study is also consistent with the long-lasting protective effects of oral contraceptive use, which seem to persist for at least 15 years after cessation of use (The Cancer and Steroid Hormone Study, 1987*a*,*b*). For cohorts born after the 1940s, the incidence risks presented a more irregular pattern, particularly for ovarian cancer, which showed a peak in risk for women born in 1950–54. However, these cohorts were still too young to be definite about their long-term risks and a marked proportion of their ovarian cancers were of germ cell origin (about 30% of ovarian cancers of known histology at ages under 30 were of germ cell origin in the present data set). The protective effect of oral contraceptive use has only been shown for epithelial ovarian cancers and its effect on other histological types is not known.

The trends in breast cancer incidence and mortality are more difficult to interpret. For women born before 1910–14, decreases in fertility at young ages paralleled the increases in breast cancer risks and may, at least in part, have been responsible for them. For women born subsequently, however, this negative relationship was replaced by a positive one. Breast cancer incidence increased for generations born from 1910–14 to 1925–29 despite their increase in fertility at young ages, and decreased steadily thereafter when the proportion of women having their first child at young ages rose but then declined.

The majority of studies have not shown any overall increase in the risk of breast cancer for women who have ever used oral contraceptives, even after a long duration of use (Malone *et al.*, 1993). Recent meta-analyses combining results from practically all studies conducted to date (Romieu *et al.*, 1990; Thomas, 1991) were also reassuring. However, studies of the effect of pill use at young ages have produced conflicting results. While some have been negative, others have shown an increased risk of premenopausal breast cancer for prolonged use of oral contraceptives at young ages (Malone *et al.*, 1993). In their meta-analyses, Romieu *et al.* (1990) and Thomas (1991) found a relative risk of about 1.5 for prolonged use at ages under 45. The UK National Case–Control Study showed a relative risk up to 1.7 for 8 years of oral contraceptive use before age 36 (UK National Case–Control Study Group, 1989). So far, cancer registration data do not suggest that breast cancer incidence at young ages might be increasing as a result of use of oral contraceptives – indeed their risks to date are lower than those of previous generations. Although an effect might be seen in the future, particularly if the latent period is long, at present it is a decrease not an increase in risk that needs explanation. It is possible that changes in menarcheal age and dietary fat intake might have been, at least in part, responsible for this decline. It has also been suggested that, like pregnancy, oral contraceptive use might have a biphasic effect on the subsequent risk of breast cancer – an early transitory adverse effect and a long-term beneficial effect (Wingo *et al.*, 1991; Beral and Reeves, 1993). A reanalysis of data from the Cancer and Steroid Hormone Study (Wingo *et al.*, 1991) showed results compatible with this hypothesis. The data presented here are consistent with a long-term beneficial effect of oral contraceptive use on the risk of breast cancer, but further individual-based studies are required to test this hypothesis. The timing of the decline in breast cancer risks and the parallels with ovarian and endometrial cancer trends are, nevertheless, striking.

**Acknowledgements**

We thank the Cancer Research Campaign for funding this work and the Office of Population Censuses and Surveys for provision of the data. The authors are members of the Epidemiological Monitoring Unit, which is funded by the Medical Research Council.

**Note added in proof**

We have now received from OPCS breast cancer incidence data for the years 1988–89. There was a sudden marked increase in the number of breast cancer registrations in 1987 which affected all age groups. This rise was restricted to the Thames Cancer Registry and it might reflect better ascertainment of this Registry since its amalgamation with the north-west and north-east regions in 1985.

### References

ALDERSON M AND DONNAN S. (1978). Hysterectomy rates and their influence upon mortality from carcinoma of the cervix. *J. Epidemiol Commun. Health*, **32,** 175–177.

BERAL V. (1974). Cancer of the cervix: a sexually transmitted infection? *Lancet*, **1,** 1037–1040.

BERAL V AND REEVES G. (1993). Childbearing, oral contraceptive use, and breast cancer. *Lancet*, **341,** 1102.

BERAL V, FRASER P AND CHILVERS C. (1978). Does pregnancy protect against ovarian cancer? *Lancet*, **1,** 1083–1087.

BOOTH M. (1991). Aetiology and epidemiology of ovarian cancer. In *Textbook of Gynaecologic Oncology*. Blackledge GRP, Jordan JA and Shingleton HM. (eds) pp. 103–113. WB Saunders: London.

BRESLOW NE AND DAY NE. (1987). *Statistical Methods in Cancer Research*, Vol. II. *The Design and Analysis of Cohort Studies*. p. 136. International Agency for Research on Cancer: Lyon.

BUSS D. (1991). The changing household diet. In *Fifty Years of the National Food Survey 1940–1990*. Slater JM. (ed.) The proceedings of a symposium held in December 1990, London, Ministry of Agriculture Fisheries and Food. HMSO: London.

CASE RAM. (1956). Cohort analysis of mortality rates as an historical or narrative technique. *Br. J. Prev. Soc. Med.*, **10,** 1959–71.

CHAMBERLAIN J, MOSS SM, KIRKPATRICK AE, MICHELL M AND JOHNS L. (1993). National Health Service breast screening programme results for 1991-92. *Br. Med. J.*, **307,** 353–6.

COLEMAN D. (1993). Britain in Europe: international and regional comparisons of fertility levels and trends. In *New Perspectives on Fertility in Britain*. Ni Bhrolchain M. (ed.) Studies on Medical and Population Subjects, No. 55. HMSO: London.

DANN TC AND ROBERTS DF. (1993). Menarcheal age in University of Warwick young women. *J. Biosoc. Sci.*, **25,** 531–538.

DEPARTMENT OF HEALTH AND SOCIAL SECURITY AND OFFICE OF POPULATION CENSUSES AND SURVEYS. (1970). *Report on Hospital In-patient Enquiry for the year 1967*, Part I, *Tables*. HMSO: London.

DEPARTMENT OF HEALTH AND SOCIAL SECURITY, OFFICE OF POPULATION CENSUSES AND SURVEYS AND WELSH OFFICE. (1981). *Hospital In-patient Enquiry, 1978, Main Tables*, Series MB4 No. 12. HMSO: London.

ELWOOD JM, COLE P, ROTHMAN KJ, KAPLAN SD. (1977). Epidemiology of endometrial cancer. *J. Natl Cancer Inst.*, **59,** 1055–1060.

GENERAL REGISTER OFFICE. (1960). *Registrar General's Statistical Review of England and Wales for the year 1959*, Part III, *Commentary*. HMSO: London.

GLASS DV AND GREBENIK E. (1954). *The Trend and Pattern of Fertility in Great Britain. A Report of the Family Census of 1946, Papers of the Royal Commission on Population*, Vol. VI. HMSO: London.

GREAVES JP AND HOLLINGSWORTH DF. (1966). Trends in food consumption in the United Kingdom. *World Rev. Nutr. Dietetics*, **6,** 34–89.

HENDERSON BE, CASAGRANDE JT, PIKE MC, MACK T, ROSARIO I AND DUKE A. (1983). The epidemiology of endometrial cancer in young women. *Br. J. Cancer*, **47,** 749–756.

HILDRETH NG, KELSEY JL, LIVOLSI VA, FISCHER DB, HOLFORD TR, MOSTOW ED, SCHWARTZ PE AND WHITE C. (1981). An epidemiologic study of epithelial carcinoma of the ovary. *Am. J. Epidemiol.*, **114,** 398–405.

MCKINLAY S, JEFFREYS M AND THOMPSON B. (1972). An investigation of age at menopause. *J. Biosoc. Sci.*, **4,** 161–173.

MACMAHON B, COLE P, LIN TM, LOWE CR, MIRRA AP, RAVNIHAR B, SALBER EJ, VALAORAS VG AND YUASA S. (1970). Age at first birth and breast cancer risk. *Bull. WHO*, **43,** 209–221.

MALONE KE, DALING JR AND WEISS NS. (1993). Oral contraceptives in relation to breast cancer. *Epidemiol. Rev.*, **15,** 80–97.

MILLER AB, BERRINO F, HILL M, PIETINEN P, RIBOLI E AND WAHRENDORF J. (1994). Diet in the aetiology of cancer: a review. *Eur. J. Cancer*, **30A,** 207–220.

OFFICE OF POPULATION CENSUSES AND SURVEYS. (1972). *The Registrar General's Statistical Review of England and Wales for the years 1970*, Part II, *Tables, Population*. HMSO: London.

OFFICE OF POPULATION CENSUSES AND SURVEYS. (1974). *The Registrar General's Statistical Review of England and Wales for the years 1972*, Part II, *Population*. HMSO: London.

OFFICE OF POPULATION CENSUSES AND SURVEYS. (1978). *Demographic Review. A Report on Population in Great Britain*, Series DR No. 1. HMSO: London.

OFFICE OF POPULATION CENSUSES AND SURVEYS. (1992*a*). *Birth Statistics. Review of the Registrar General on births and patterns of family building in England and Wales, 1989*. Series FM1 No. 18. HMSO: London.

OFFICE OF POPULATION CENSUSES AND SURVEYS. (1992*b*). *Population Trends*, Vol. 68. HMSO: London.

OFFICE OF POPULATION CENSUSES AND SURVEYS & GENERAL REGISTER OFFICE FOR SCOTLAND. (1993). *1991 Census. Historical Tables*. HMSO: London.

PIKE MC, KRAILO MD, HENDERSON BE, CASAGRANDE JT AND HOEL DG. (1983). 'Hormonal' risk factors, 'breast tissue age' and the age-incidence of breast cancer. *Nature*, **303,** 767–70.

PRENTICE RL AND THOMAS DB. (1987). On the epidemiology of oral contraceptives and disease. *Adv. Cancer Res.*, **49,** 285–401.

REGISTRAR GENERAL. (1947). *The Registrar General's Statistical Review of England and Wales for the Years 1938 and 1939. Text*. HMSO: London.

ROMIEU I, BERLIN JA AND COLDITZ G. (1990). Oral contraceptives and breast cancer. Review and meta-analysis. *Cancer*, **66,** 2253–2263.

ROYAL COLLEGE OF GENERAL PRACTITIONERS' MANCHESTER RESEARCH UNIT. (1986). New oral contraception study: pilot trial report. *J.R. Coll. Gen. Pract.*, **36,** 545–546.

SWERDLOW AJ. (1987). 150 years of Registrar General's medical statistics. *Pop. Trends*, **48,** 20–26. HMSO: London.

SWERDLOW AJ AND DOS SANTOS SILVA I. (1993). *Atlas of Cancer Incidence in England and Wales, 1968–85*. Oxford: Oxford University Press.

SWERDLOW AJ, DOUGLAS AJ, VAUGHAN HUDSON G AND VAUGHAN HUDSON B. (1993). Completeness of cancer registration in England and Wales: an assessment based on 2,145 patients with Hodgkin's disease independently registered by the British National Lymphoma Investigation. *Br. J. Cancer*, **67,** 326–329.

THE CANCER AND STEROID HORMONE STUDY OF THE CENTERS FOR DISEASE CONTROL AND THE NATIONAL INSTITUTE OF CHILD HEALTH AND HUMAN DEVELOPMENT. (1987*a*). The reduction in risk of ovarian cancer associated with oral contraceptive use. *N. Engl. J. Med.*, **316,** 650–655.

THE CANCER AND STEROID HORMONE STUDY OF THE CENTERS FOR DISEASE CONTROL AND THE NATIONAL INSTITUTE OF CHILD HEALTH AND HUMAN DEVELOPMENT. (1987*b*). Combination oral contraceptive use and the risk of endometrial cancer. *JAMA*, **257,** 796–800.

THE CENTERS FOR DISEASE CONTROL CANCER AND STEROID HORMONE STUDY. (1983). Oral contraceptive use and the risk of ovarian cancer. *JAMA*, **249,** 1596–1599.

THOMAS DB. (1991). Oral contraceptives and breast cancer: review of the epidemiologic literature. *Contraception*, **43,** 597–642.

THOROGOOD M AND VESSEY MP. (1990). Trends in use of oral contraceptives in Britain. *Br. J. Fam. Planning*, **16,** 41–53.

UK NATIONAL CASE-CONTROL STUDY GROUP. (1989). Oral contraceptive use and breast cancer risk in young women. *Lancet*, **1,** 973–82.

WERNER B AND CHALK S. (1986). Projections of first, second, third and later births. *Pop. Trends*, **46,** 26–34.

WINGO PA, LEE NC, ORY HW, BERAL V, PETERSON HB AND RHODES P. (1991). Age-specific differences in the relationship between oral contraceptive use and breast cancer. *Am. J. Obstet. Gynecol.*, **78,** 161–170.

WISEMAN RA. (1984). Absence of correlation between oral contraceptive usage and cardio-vascular mortality. *Int. J. Fertil.*, **29,** 198–208.

WORLD HEALTH ORGANIZATION. (1957, 1967, 1977). *Manual of the International Statistical Classification of Diseases, Injuries, and Causes of Death*, seventh, eight and ninth revisions. World Health Organisation: Geneva.

WYSHAK G AND FRISCH RE. (1982). Evidence for a secular trend in age of menarche. *N. Engl. J. Med.*, **306,** 1033–1035.